# Cytomegalovirus reactivation in patients with large B-cell lymphoma treated with chimeric antigen receptor T-cell therapy

**DOI:** 10.1007/s12185-025-04023-y

**Published:** 2025-06-17

**Authors:** Kenta Hayashino, Keisuke Seike, Taro Masunari, Risa Hashida, Satoshi Oka, Yuki Fujiwara, Toshiki Terao, Wataru Kitamura, Hiroki Kobayashi, Chihiro Kamoi, Takumi Kondo, Hideaki Fujiwara, Noboru Asada, Daisuke Ennishi, Keiko Fujii, Nobuharu Fujii, Yoshinobu Maeda

**Affiliations:** 1 Department of Hematology and Oncology, Okayama University, 2-5-1 Shikata-cho, Kita-ku, Okayama, Okayama 700-8558 Japan; 2 Department of Hematology, Oncology and Respiratory Medicine, Okayama University Graduate School of Medicine, Dentistry and Pharmaceutical Sciences, Okayama, Japan; 3 Department of Hematology, Chugoku Central Hospital, 148-13, Kamiiwanari, Miyuki-cho, Fukuyama, 720-0001 Japan; 4 Division of Hematology, Ehime Prefectural Central Hospital, 83, Kasuga-machi, Matsuyama, 790-0024 Japan; 5 Department of Hematology and Blood Transfusion, Kochi Health Science Center, Ike, Kochi 2125-1781-8555 Japan; 6 Department of Hematology and Oncology, Japanese Red Cross Society Himeji Hospital, 1-12-1, Shimoteno, Himeji, 670-8540 Japan; 7 Division of Transfusion and Cell Therapy, Okayama University Hospital, 2-5-1 Shikata, OkayamaOkayama-shi, Japan; 8 Center for Comprehensive Genomic Medicine, Okayama University Hospital, 2-5-1 Shikata, Okayama-shi, Okayama Japan; 9 Division of Clinical Laboratory, Okayama University Hospital, Okayama, 2-5-1 Shikata, Okayama-shi, Japan

**Keywords:** Cytomegalovirus reactivation, Large B-cell lymphoma, CAR T-cell therapy, Hypogammaglobulinemia

## Abstract

Chimeric antigen receptor (CAR) T-cell therapy has improved outcomes of relapsed and/or refractory large B-cell lymphoma (r/r LBCL). However, its off-tumor effects result in severe prolonged humoral immune deficiency. Cytomegalovirus (CMV) is a latent virus that can be life-threatening in immunosuppressed patients. In the setting of CAR T-cell therapy, Asian race is a risk factor for clinically significant CMV infection. However, the effect of CAR T-cell therapy on CMV reactivation in Japanese patients remains unclear. Previous reports used polymerase chain reaction (PCR), but we used the pp65 antigenemia assay to retrospectively investigate long-term effects in patients with r/r LBCL. The study included 46 patients. Nine (19.6%) developed CMV reactivation, with a median onset of 13 days. Six of these patients received preemptive therapy, and none developed CMV end-organ disease. Primary refractory disease, grade 2–4 cytokine release syndrome, and high-dose corticosteroids were risk factors for CMV reactivation. Long-term follow-up showed that CMV reactivation rarely occurred later than 28 days post-infusion. Our study using the pp65 antigenemia assay showed a similar incidence of CMV reactivation, onset, and risk factors to those in the previous reports using PCR.

**Supplementary Information:**

The online version contains supplementary material available at 10.1007/s12185-025-04023-y.

## Introduction

Chimeric antigen receptor (CAR) T-cell therapy has significantly altered the treatment of patients with relapsed and/or refractory large B-cell lymphoma (r/r LBCL). CAR T-cell therapy for patients with r/r LBCL induces B-cell aplasia, because CD19 are expressed in both lymphoma and normal B-cells and cause severe and prolonged humoral immune deficiency. Furthermore, immune function is affected by the original disease, chemotherapy before CAR T-cell therapy, lymphodepletion chemotherapy, immunosuppressive therapy for cytokine release syndrome (CRS) and/or immune effector cell-associated neurotoxicity syndrome (ICANS), and treatment after CAR T-cell therapy failure. Patients treated with CAR T-cell therapy, in particular, are prone to infection because of severe cellular and humoral immune deficiency. Some reports have described that herpes simplex virus, varicella-zoster virus, and hepatitis B virus (HBV) can be reactivated, and prophylactic acyclovir or valaciclovir and regular monitoring for HBV are recommended in treatment of CAR-T cell therapy [1–5].

Cytomegalovirus (CMV) is a potentially life-threatening latent virus that can react in patients who are immunosuppressed, inducing asymptomatic viremia, pneumonia, gastrointestinal diseases, hepatitis, retinitis, and encephalitis [6, 7]. In the setting of treatment for hematological malignancies, the association between allogeneic stem cell transplantation (allo-SCT) and CMV reactivation has been well explored. After allo-SCT, 40–45% of patients experienced CMV reactivation, and it was associated with increased non-relapse mortality (NRM). Prophylactic letermovir improved the proportion of patients with clinically significant CMV infection [8, 9]. However, in non-transplant settings, the association between treatments for hematological malignancies and CMV reactivation remains controversial. The rate of CMV infection/reactivation ranges from 2 to 67%, and extensive prophylaxis or preemptive treatments are not generally recommended [10]. Treatment with bendamustine, rituximab, and high-dose corticosteroids, disease progression, and poor performance status are risk factors for CMV reactivation in these patients [10–13].

Polymerase chain reaction (PCR) is commonly used to monitor CMV reactivation, because it is more sensitive than pp65 antigenemia assay and allows the reactivation to be treated in its early phase [14, 15]. Some previous studies have described CMV infection/reactivation following CAR T-cell therapy, and all of them were evaluated using PCR assays. In these studies, patients were evaluated using PCR assays from various manufacturers using various definitions of CMV-PCR positivity. Therefore, the association between CAR T-cell therapy and CMV reactivation is difficult to understand [16–21] (Table 1). In contrast, CMV reactivation is still monitored using pp65 antigenemia assays in many countries, including Japan, since it is rapid, easy to perform, and low cost [14, 22, 23]. However, the association between CMV reactivation and CAR T-cell therapy using pp65 antigenemia assay has not been clarified. In addition, in CAR T-cell therapy settings, the Asian race is associated with clinically significant CMV infection [16]; however, to the best of our knowledge, no prior reports have described CMV reactivation in Japanese patient cohort. Furthermore, humoral immune deficiency is prolonged (> 1 year) in the majority of the patients who received CAR T-cell therapy [24, 25], the frequency and duration of monitoring patients undergoing CAR T-cell therapy remains unclear.

**Table 1:** Previous and present reports of chimeric antigen receptor T-cell therapy and cytomegalovirus infection

	1	2	3	4	5	6	Present report
References	[16]	[17]	[18]	[19]	[20]	[21]	
Number of patients, *n*	230	65	60	95	72	95	46
Country	USA	USA	Israel	USA	USA	Spain	Japan
Age, years (median)	60	63	69	62	64	61	58
Sex, male, *n* (%)	159 (69)	40 (62)	31 (52)	64 (67)	41 (57)	60 (63)	21 (46)
CAR-T							
Tisa-cel	23 (10)	27 (42)	44 (73)	7 (7)	3 (4)	38 (40)	42 (91)
Liso-cel	0	5 (8)	0	2 (2)	21 (29)	0	2 (4)
Axi-cel	205 (89)	32 (49)	16 (27)	67 (71)	15 (21)	26 (27)	2 (4)
Other	2 (1)	1 (2)	0	19 (20)	33 (46)	31 (33)	0
Disease							
NHL	219 (95)	65 (100)	60 (100)	88 (93)	55 (76)	95 (100)	46 (100)
B-ALL	11 (5)	0	0	3 (3)	3 (4)	0	0
MM	0	0	0	4 (4)	14 (19)	0	0
CMV reactivation, *n* (%)	51 (22)	14 (22)	10 (17)	31 (33)	19 (26)	41 (43)	9 (20)
CMV end-organ disease, *n* (%)	7 (3)	1 (2)	0	0	0	0	0
Median time of CMV reactivation, days from infusion	17	21	NA	19	NA	21	13
Treatment for CMV, *n* (%)	15 (7)	10 (15)	6 (10)	10 (11)	5 (7)	7 (7)	6 (13)
Treatment threshold	CMV-PCR > 500 IU/mL	Treating physician judgment (all cases PCR > 1000 IU/mL	PCR > 1000 copies/mL	CMV-PCR > 200 IU/mL	CMV-PCR > 150 IU/mL	CMV-PCR > 10,000 IU/mL	Treating physician judgment (All cases 6 ≥/50000 PBL)
Define of CMV reactivation/infection	PCR positive	PCR > 400 IU/mL	PCR > 1000 copies/mL	PCR positive	PCR > 50 IU/mL	PCR positive	CMV antigenemia positive
PCR assay	NA	NA	NA	Roche	Abbott	AltoStar	NA
Risk factor for CMV infection	Asian and Middle Eastern race, corticosteroid use, high LDH	Older age, CMV-seropositive	NA	Severe CRS, corticosteroid use	BCMA-CART, Corticosteroid > 3 days, number of prior treatment regimens	Corticosteroid use	Primary refractory, Grade 2–4 CRS, high dose of corticosteroid

ALL, acute lymphoblastic leukemia; Axi-cel, axicabtagene ciloleucel; BCMA, B-cell maturation antigen; CAR T, chimeric antigen receptor T; CMV, cytomegalovirus; CRS, cytokine release syndrome; LDH, lactate dehydrogenase; Liso-cel, lisocabtagene maraleucel; MM, multiple myeloma; NA, not available; NHL, non-Hodgkin Lymphoma; PBL, peripheral blood leukocytes; PCR, polymerase chain reaction; Tisa-cel, tisagenlecleucel

The present study aimed to determine the association between CAR T-cell therapy and CMV reactivation, including risk factors and the duration of pp65 antigenemia monitoring, in Japanese patients. To this end, we retrospectively analyzed patients treated with CAR T-cell therapy at our institution over long-term follow-up.

## Materials and methods

### Patient characteristics

This retrospective study included patients aged ≥ 18 years with r/r LBCL who underwent commercial CAR T-cell therapy at Okayama University Hospital between December 2019 and July 2024. We utilized data from medical records to analyze the general characteristics of the patients and their disease. All patients underwent at least one pp65 antigenemia assay. LBCL, including the following diagnoses, based on the 4th edition of the World Health Organization’s Classification [26]: diffuse large B-cell lymphoma-not otherwise specified (DLBCL-NOS) and specific subtypes of DLBCL, including primary central nervous system lymphoma, primary mediastinal B-cell lymphoma, transformed indolent B-cell lymphoma, and immunodeficiency-associated DLBCL. The clinical stage was defined using the Ann Arbor system. The protocol for this study was approved by the Institutional Review Board of Okayama University Hospital (#2405-051) and conducted in accordance with the tenets of the Declaration of Helsinki. Information about this study was disclosed on a website which provided an opt-out option. None of the participants opted out based on the study documents available to them on the aforementioned website.

### Study definitions and endpoints

CMV reactivation and CMV end-organ disease were defined based on the previous reports [27]. We defined CMV reactivation as ≥ 1 cell/50,000 peripheral blood leukocytes (PBLs). Based on the criteria of a previous clinical trial, clinically significant CMV infection (CS-CMVi) was defined as CMV disease or CMV viremia leading to preemptive treatment [9]. During hospitalization, CMV monitoring was generally performed weekly after CAR T-cell infusion. In cases of detectable pp65 antigenemia or CMV-related symptoms (e.g., cytopenia, fever, and pneumonia), intensified evaluation was performed. The timing of treatment was determined at the physician’s discretion, considering the level or kinetics of antigenemia (e.g., a rapid increase), although no numerical threshold was established. CRS and ICANS were graded according to the American Society for Transplantation and Cellular Therapy grading system [28]. Other adverse events were graded as defined by the Common Terminology Criteria for Adverse Events v. 5.0. The cumulative corticosteroid dosages used for CRS and/or ICANS were calculated, with the strength of each corticosteroid converted to prednisolone (PSL) equivalent. Based on the previous reports, hydrocortisone 100 mg, PSL 25 mg, methylprednisolone 20 mg, and dexamethasone 4 mg were considered equivalent doses [29]. The standard dose of tocilizumab was 8 mg/kg.

Patient performance status (PS) was classified as defined by the Eastern Cooperative Oncology Group. Baseline laboratory values, such as lactate dehydrogenase (LDH) and lymphocyte count, were defined as those obtained just prior to the administration of lymphodepleting chemotherapy. The immunoglobulin G (IgG), B-cell, CD4+ T-cell, and CD8+ T-cell counts utilized were the lowest values obtained when examined several times during the same period. A high CAR-HEMATOTOX score was defined as greater than one points, according to a previous report [30]. All patients received prophylactic sulfamethoxazole-trimethoprim (sulfamethoxazole 400 mg and trimethoprim 80 mg) once daily and valaciclovir 500 mg twice daily from lymphodepleting chemotherapy to 5 weeks after infusion. From 5 weeks after infusion, valaciclovir was switched to acyclovir 200 mg orally once daily. Patients received intravenous immunoglobulin to support general infection prophylaxis, and CMV-specific intravenous immunoglobulin was not administered. The treatment response was based on the International Working Group response criteria [31]. The disease status prior to CAR T-cell infusion was classified as follows: complete response (CR), any CR; relapse, non-CR after initially achieving CR; and primary refractory, failure to achieve CR after starting treatment. The primary endpoint of this study was the cumulative incidence of CMV reactivation using pp65 antigenemia assay.

### Statistical analysis

Categorical and continuous variables were analyzed using Fisher’s exact test and the Mann–Whitney *U* test, respectively. The cumulative incidence of CMV reactivation and NRM was calculated using Gray’s test. CMV reactivation and death or relapse before CMV reactivation were the competing risks. To identify the factors associated with CMV reactivation, univariate analysis was performed using the Fine–Gray proportional hazards regression. Progression-free survival (PFS) was defined as the time from CAR T-cell infusion to relapse, disease progression, death, or last follow-up. Overall survival (OS) was defined as the time from CAR T-cell infusion to the last follow-up or death. NRM was defined as the time to death without relapse or disease progression. OS and PFS were estimated using the Kaplan–Meier method, and group comparisons were performed using the log-rank test. Survival outcomes were evaluated using univariate and multivariate Cox proportional hazards analyses. NRM and relapse or progression were treated as competing risks. Statistical significance was set at *p* < 0.05. All statistical analyses were performed using EZR software (Ver. 1.61), a graphical user interface for R version 4.2 (The R Foundation for Statistical Computing, Vienna, Austria) [32].

## Results

### Patient characteristics

A total of 46 patients underwent CAR T-cell therapy and pp65 antigenemia assay at least once; their characteristics are summarized in Table 2. All the patients were Japanese. The majority of the patients received tisagenlecleucel (*n* = 42), followed by axicabtagene ciloleucel (*n* = 2) and lisocabtagene maraleucel (*n* = 2). None of the patients received prophylaxis for CMV reactivation in conjunction with CAR T-cell infusion.

**Table 2:** Patient characteristics in the study

	Total (*n* = 46)	CMV reactivation (*n* = 9)	Non-CMV reactivation (*n* = 37)	*p*
Median age, years (range)	58 (42–72)	61 (53–69)	58 (42–72)	0.59
Sex, *n* (%)				0.71
Male	21 (45.7)	5 (55.6)	16 (43.2)	
Female	25 (54.3)	4 (44.4)	21 (56.8)	
CMV-IgG, *n* (%)				0.56
Positive	27 (58.7)	6 (66.7)	22 (59.5)	
Negative	4 (8.7)	0	4 (10.8)	
NA	15 (32.6)	3 (33.3)	11 (29.7)	
Prior history of CMV infection, *n* (%)	6 (13.0)	3 (33.3)	3 (8.1)	0.08
Disease histology, *n* (%)				0.38
DLBCL-NOS	28 (60.9)	6 (66.7)	22 (59.5)	
Transformed	11 (23.9)	3 (33.3)	8 (21.6)	
Other	7 (15.2)	0	7 (18.9)	
CAR-T products, *n* (%)				0.60
Tisa-cel	42 (91.3)	8 (88.9)	34 (91.9)	
Liso-cel	2 (4.3)	0	2 (5.4)	
Axi-cel	2 (4.3)	1 (11.1)	1 (2.7)	
Prior history of auto-SCT, *n* (%)	19 (41.3)	1 (11.1)	18 (48.6)	0.21
Disease status, *n* (%)				0.0068
CR	9 (19.6)	0	9 (24.3)	
Primary refractory	15 (32.6)	7 (77.8)	8 (21.6)	
Relapse	22 (47.8)	2 (22.2)	20 (54.1)	
Median number of prior regimens, *n* (range)	4 (2–7)	5 (4–7)	3.5 (2–6)	0.0029
PS, *n* (%)				0.35
0–1	37 (80.4)	6 (66.7)	31 (83.8)	
2–	9 (19.6)	3 (33.3)	6 (16.2)	
CAR-HEMATOTOX, *n* (%)				0.0062
High	27 (58.7)	9 (100)	18 (48.6)	
Low	19 (41.3)	0	19 (51.4)	
Median LDH (U/L) (range)	228.5 (136–1344)	340 (217–1344)	224.5 (136–1250)	0.0033
Median Ly (/mL) (range)	515 (100–2150)	370 (150–1340)	520 (100–2150)	0.20
Median B-cell (/mL) (range)	0 (0–0)	0 (0–0)	0 (0–0)	NA
Median CD4 + T-cell (/mL) (range)	134.5 (45–482)	147 (45–482)	102 (102–102)	0.83
Median CD8 + T-cell (/mL) (range)	383 (74–759)	563 (74–759)	163 (163–163)	0.83
Lymphodepleting chemotherapy, n (%)				0.21
FLU/CY	41 (89.1)	8 (88.9)	33 (89.2)	
Other	5 (10.9)	1 (11.1)	4 (10.8)	
CRS, *n* (%)				0.00036
Grade 0–1	30 (65.2)	1 (11.1)	29 (78.4)	
Grade 2–4	16 (34.8)	8 (88.9)	8 (21.6)	
ICANS, *n* (%)				0.57
Grade 0–1	42 (91.3)	9 (100)	33 (89.2)	
Grade 2–4	4 (8.7)	0	4 (10.8)	
Corticosteroid use, *n* (%)	31 (67.4)	6 (66.7)	24 (64.9)	1.0
Cumulative dose of PSL > 1000 mg, *n* (%)	6 (13.0)	3 (33.3)	3 (8.1)	0.079
Tocilizumab use, *n* (%)	39 (84.8)	9 (100)	30 (81.1)	0.32
Cumulative dose of tocilizumab, median (range)	2 (0–4)	2 (1–4)	2 (0–4)	0.73
Number of pp65 antigenemia assays, median (range)	4 (1–42)	6 (1–42)	4 (1–29)	0.30

Axi-cel, axicabtagene ciloleucel; auto-SCT, autologous stem cell transplantation, CAR-T, chimeric antigen receptor-T; CMV, cytomegalovirus; CR, complete response; CRS, cytokine release syndrome; CY, cyclophosphamide; DLBCL-NOS, diffuse large B-cell lymphoma-not otherwise specified; FLU, fludarabine; ICANS, immune effector cell-associated neurotoxicity syndrome; LDH, lactate dehydrogenase; Liso-cel, lisocabtagene maraleucel; Ly, lymphocyte; NA, not available; PS, performance status; PSL, prednisolone; Tisa-cel, tisagenlecleucel

The patient characteristics were compared between the CMV reactivation (*n* = 9) and non-CMV reactivation (*n* = 37) groups. The ratios of the patients with history of CMV reactivation were higher in CMV reactivation group (*p* = 0.08). The majority of patients in the CMV reactivation group were primary refractory, and the median number of prior regimens was higher in the CMV reactivation group than non-CMV reactivation group (median: 5 vs. 3.5, *p* = 0.0029). All patients in the CMV reactivation group had high CAR-HEMATOTOX scores (*p* = 0.0062). The CMV reactivation group had significantly higher LDH level than the non-CMV reactivation group (median: 340 vs. 224.5 U/L, *p* = 0.0033). The incidence of grade 2–4 CRS in the CMV reactivation group was markedly higher than that in the non-CMV reactivation group (*p* = 0.00036). The rates of corticosteroid and tocilizumab use, and cumulative tocilizumab dose were similar in both groups; however, the proportion of patients treated with > 1000 mg of PSL equivalent for CRS and/or ICANS was higher in the CMV reactivation group (*p* = 0.079). There were no significant differences between the CMV and non-CMV reactivation groups in terms of age, sex, CMV-seropositive, disease histology, type of CAR T-cell products, prior history of autologous stem cell transplantation (auto-SCT), PS, lymphocyte, B-cell, CD4+ T-cell, and CD8+ T-cell count, lymphodepleting chemotherapy, grade 2–4 ICANS, and number of pp65 assays.

To determine the impact of neutropenia on pp65 antigenemia assays, the neutrophil kinetics during CAR T-cell therapy are shown in Supplemental Fig. 1. The neutrophil counts were gradually decreased following lymphodepleting chemotherapy, and neutropenia persisted throughout the observation period. A total of 41 patients (89.1%) experienced grade 4 neutropenia, with a median duration of 8 days (range 1–25 days). Furthermore, neutrophil count fell below 200/mL in 30 patients (65.2%), with a median duration of 4.5 day (range 1–18 days).

**Fig. 1:**
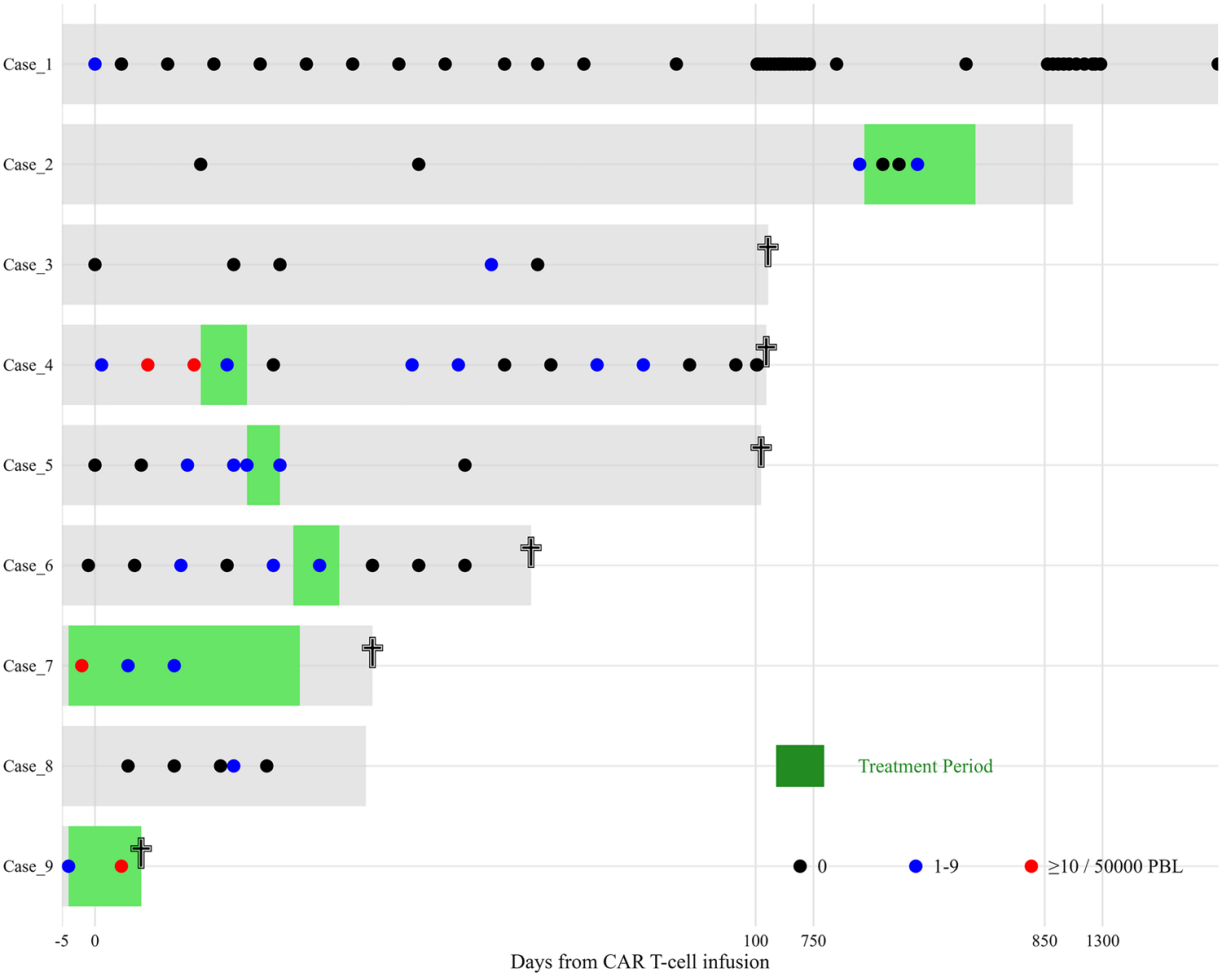
The clinical course in the patients with CMV reactivation. CAR T, chimeric antigen receptor T; CMV, cytomegalovirus; PBL, peripheral blood leukocytes

### Incidence of CMV reactivation

**Fig. 2:**
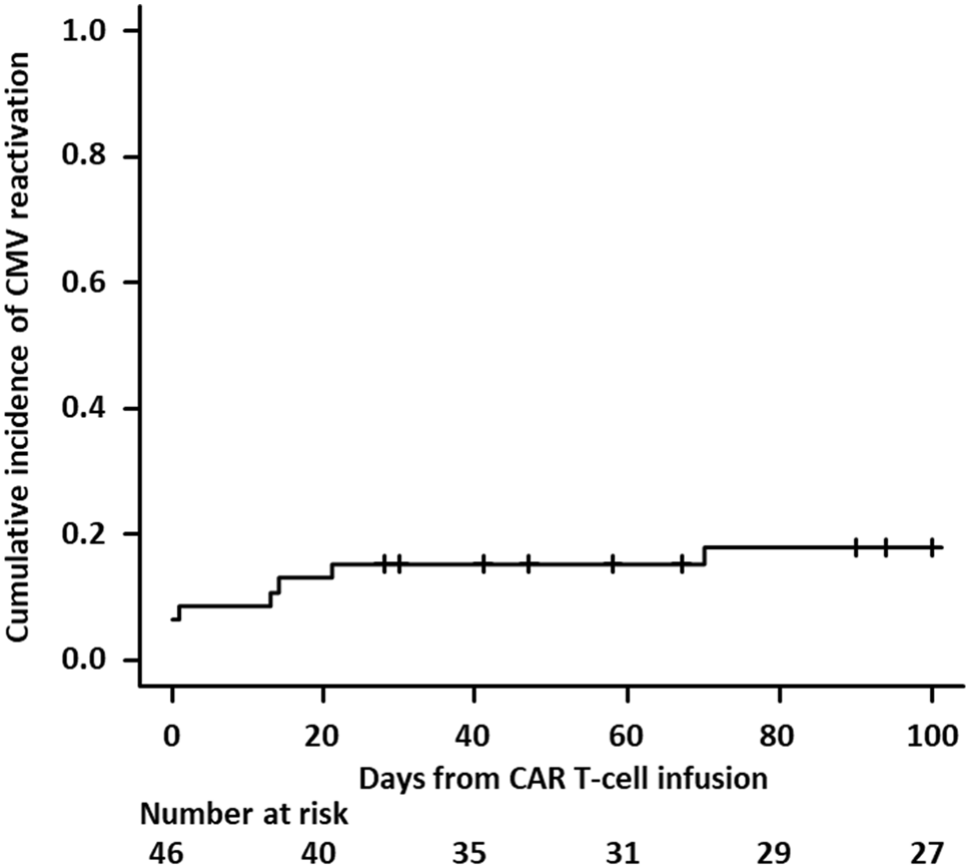
The cumulative incidence of CMV reactivation. CAR T, chimeric antigen receptor T; CMV, cytomegalovirus;

The median CMV follow-up duration was 53 (range 4–1627) days, with the duration exceeding 28 days in 28 patients, 100 days in 17 patients, and 1 year in 12 patients. The median number of pp65 antigenemia assays was 4 (range 1–42). Of the included patients, 9 (19.6%) developed CMV reactivation, but none had evidence of CMV end-organ disease. The clinical courses of the nine patients who developed CMV reactivation is shown in Fig. 1. The median time from CAR T-cell infusion to first reactivation was 13 (range − 4 to 770) days, and median duration of viremia was 10 (range 3–89) days. The cumulative incidence of CMV reactivation was 15.2% (95% confidence interval [CI] 7.6–29.3%) at day 28, and 18.0% (95% CI 9.4–33.0%) at day 100 post-infusion (Fig. 2). The pp65 antigenemia assays were negative in all patients before the initiation of lymphodepleting chemotherapy, although two patients developed CMV reactivation during lymphodepleting chemotherapy (Case_7 and Case_9).

Of the nine patients with CMV reactivation, six received preemptive therapy with ganciclovir or valganciclovir, with a median duration of 8.5 (range 4–22) days. At the start of preemptive therapy, the median number of positive cells was 22.5 (range 6–95) cells/50,000 PBLs. In contrast, CMV reactivation resolved in three patients without any intervention. The pp65 antigenemia assays in these patients revealed 1 positive cell/50,000 PBLs, while the subsequent assays were negative.

The CMV reactivation group showed worse 1-year PFS (25.9% vs. 54.2%; 95% CI 3.9–57.0% vs. 36.4–68.9%; *p* = 0.10) and significantly worse 1-year OS than the non-CMV infection group (25.4% vs. 73.5%; 95% CI 3.8–56.4% vs. 55.2–85.3%; *p* = 0.0092) (Supplemental Fig. 2a, b). In univariate analysis of PFS, CMV reactivation was not significantly associated with a poor prognosis (hazard ratio [HR] 2.2; 95% CI 0.84–5.5, *p* = 0.11). In contrast, CMV reactivation was significantly associated with a poor OS in univariate analysis (HR 3.4; 95% CI 1.3–9.1, *p* = 0.014), however, the statistical significance of this association was lost in multivariate analysis (Supplemental Table 1). There were no cases of NRM in either group.

### Risk factors of CMV reactivation

Univariate analyses indicated that the following were risk factors for CMV reactivation within 28 days from infusion: primary refractory, grade 2–4 CRS, and high-dose corticosteroid (> 1000 mg of PSL equivalent for the treatment of CRS and/or ICANS). In contrast, advanced age (> 60 years), prior history of CMV reactivation, prior history of auto-SCT, number of prior regimens > 4, lymphopenia (lymphocyte count < 500/µL), high serum levels of LDH (LDH > upper limit of normal), and hypogammaglobulinemia (IgG < 500 mg on days 0–28) were not associated with CMV reactivation (Table 3).

**Table 3:** Univariate analysis of risk factor for cytomegalovirus reactivation within 28 days

	HR	95% CI	*p* Value
Age > 60	3.1	0.61–15.7	0.17
Prior history of CMV reactivation	3.2	0.65–16.0	0.15
Prior history of auto-SCT	0.22	0.028–1.7	0.14
Primary refractory	6.1	1.3–28.8	0.021
Number of prior regimens > 4	4.1	0.96–17.3	0.056
LDH > ULN	4.2	0.53–32.8	0.18
Lymphocyte counts < 500/µL	1.7	0.41–7.1	0.47
CRS Grade 2–4	12.8	1.5–109.4	0.02
Use of PSL > 1000 mg	5.7	1.4–23.11	0.014
IgG day0-28 < 500 mg/dL	1.6	0.38–7.0	0.51

auto-SCT, autologous stem cell transplantation; CI, confidence intervals; CMV, cytomegalovirus; CRS, cytokine release syndrome; HR, hazard ratio; IgG, immunoglobulin G; LDH, lactate dehydrogenase; PSL, prednisolone; ULN, upper limit of normal

**Fig. 3:**
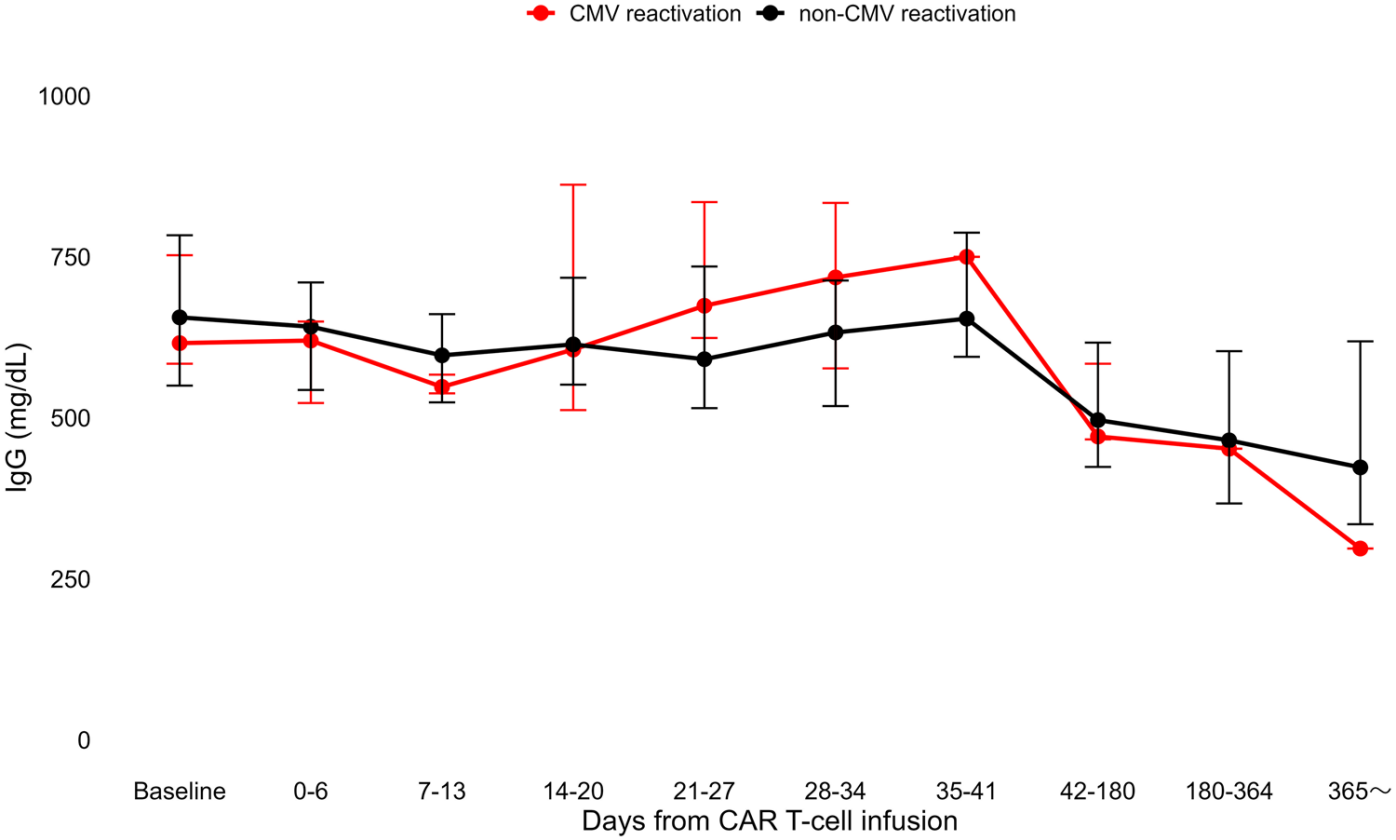
Comparison of the kinetics of IgG levels (median ± IQR) in the CMV reactivation group and non-CMV reactivation group. CAR T, chimeric antigen receptor T; CMV, cytomegalovirus; IgG, immunoglobulin G; IQR, interquartile range

We also compared the kinetics of median serum IgG levels before and after CAR T-cell therapy in the CMV and non-CMV reactivation within 28 days from infusion groups. The median IgG level showed no significant difference between the two groups (Fig. 3). The frequency of intravenous immunoglobulin administration within 28 days of infusion was similar in both groups (CMV reactivation group, 71.4%; non-CMV reactivation group, 56.1%; *p* = 0.68).

### Comparison of outcomes between CS-CMVi and non-CS-CMVi

Given the clinical importance of CS-CMVi, we conducted an additional analysis comparing the CS-CMVi group (*n* = 6) and the non-CS-CMVi group (*n* = 40). Patient characteristics for both groups are summarized in Supplemental Table 2. In the CS-CMVi group, all patients had high CAR-HEMATOTOX scores. Additionally, in this group, the majority of patients were primary refractory, had received extensive prior treatment (> 4 regimens), had elevated LDH levels, and experienced grade 2–4 CRS.

Among the six patients with CS-CMVi, the median time from CAR T-cell infusion to first reactivation was 7 days (range − 4 to 770). The cumulative incidence of CS-CMVi by day 28 was 10.9% (95% CI: 4.0–21.7%) (Supplemental Fig. 3). The CS-CMVi group showed significantly worse 1-year PFS and 1-year OS than the non-CS-CMVi group (16.7% vs. 54.1%; 95% CI: 0.8–51.7% vs. 36.9–68.6%; *p* = 0.017, and 16.7% vs. 72.2%; 95% CI: 0.8–51.7 vs. 54.4–84.0%, *p* = 0.00077). Univariate analyses identified the following as risk factors for CS-CMVi: primary refractory disease, grade 2–4 CRS, and high-dose corticosteroid use (Supplemental Table 3).

### CMV reactivation in patients with long-term follow-up

Of the included patients, 28 were followed up beyond 28 days post-infusion. Characteristics of these 28 patients are summarized in Supplemental Table 4. The median CMV follow-up duration was 186 (range 30–1627) days. The median number of pp65 antigenemia assays in this cohort was 7 (range 1–42) post-infusion and 4 (range 1–37) after day 28.

Supplemental Fig. 4a shows the kinetics of the median serum IgG level at 1 month or more post-infusion. The serum IgG levels were approximately 400–500 mg/dL, and 80% (16/20) of the patients received immunoglobulin prophylaxis. We also analyzed lymphocyte kinetics. Only one patient recovered B cells during the observation period without relapse or disease progression. Supplemental Fig. 4b, c shows the kinetics of CD4+ T-cell and CD8+ T-cell. CD4+ T-cell gradually recovered from infusion, and no one experienced a CD4+ T-cell count below 50/mL.

Two patients developed a CMV reactivation later than day 28. One patient (Case_2) was infected with severe acute respiratory syndrome coronavirus 2 and developed organizing pneumonia. The patient was treated with PSL, and her CD4 + T-cell count was decreased from 92 to 61/µL. She developed an asymptomatic CMV reactivation (7 positive cell/50000 PBLs) on day 770 and was treated with valganciclovir, after which the CMV reactivation immediately improved. In another case (Case_3), the patient achieved CR following CAR T-cell therapy and was asymptomatic for CMV; however, pp65 antigenemia assay detected CMV reactivation (1/50,000 PBLs) on day 70 with CD4+ T-cell count 306/mL, which resolved spontaneously 1 week later without any treatment.

## Discussion

In the present study, we utilized pp65 antigenemia assay to determine the incidence of CMV reactivation in patients with r/r LBCL who received CAR T-cell therapy. Of the 46 included patients, 9 (19.6%) developed CMV reactivation and 6 (13.0%) received preemptive therapy, but none developed CMV end-organ disease. Only univariate analysis has been conducted owing to the small number of cases, primary refractory, grade 2–4 CRS, and high-dose corticosteroids might be risk factor for CMV reactivation. Although most patients experienced severe and prolonged B-cell aplasia and hypogammaglobulinemia, CMV reactivation rarely developed in patients more than 28 days post-infusion. In univariate analysis, CMV reactivation was significantly associated with poor OS; however, in multivariate analysis, the statistical significance of this association was lost in multivariate analysis and no patient experienced NRM.

Previous studies have described that the prevalence of CMV infection/reactivation ranged from 17 to 43% (Table 1). The median time of CMV reactivation is 17–21 days, and 7–15% of patients received preemptive therapy. High tumor burden, more prior antitumor regimens, severe CRS, and corticosteroid use were risk factor CMV reactivation [16–19, 21, 33] (Table 1). In this study, all of the patients were Japanese and were evaluated using pp65 antigenemia assays. We defined CMV reactivation as ≥ 1 cell/50,000 PBLs, and 9 patients (19.6%) developed CMV reactivation, which is in accordance with the aforementioned analysis. Additionally, the median onset of CMV reactivation, treatment ratio, and risk factors for CMV reactivation were similar to those reported previously. In addition, the prevalence of CS-CMVi in our study was consistent with a previous report. The risk factors for CS-CMVi were similar to those for CMV reactivation, and CS-CMVi was also associated with poor survival outcomes [16, 34]. Notably, in this study, all patients in the CMV reactivation group had high CAR-HEMATOTOX scores. To our knowledge, only one prior report has described the association between CS-CMVi and CAR-HEMATOTOX [33]. However, in that report, there was no significant difference between the CS-CMVi and non-CS-CMVi groups. Moreover, the cohort included patients with acute lymphoblastic leukemia, chronic lymphocytic leukemia, and multiple myeloma, and the proportion of high-risk patients was lower than that in our study (30.6% vs. 58.7%). Therefore, direct comparison is difficult.

CAR T-cell therapy can result in cellular and humoral immune deficiencies. In CAR T-cell therapy settings, cellular immune deficiency is transient, because it primarily depends on lymphodepletion chemotherapy or immunosuppressive therapy for CRS and/or ICANS. Cellular immunity plays a crucial role in controlling CMV reactivation [35, 36]. A number of CD4 + T cells below 200/µL is a risk factor for severe infection, while 50/µL is a risk factor for CMV reactivation in acquired immunodeficiency syndrome [37, 38]. A prior report described that CMV-specific cell-mediated immunity reached a nadir 2 weeks after CAR T-cell infusion and recovered to baseline levels by week 4 [33]. Indeed, in this study, high-dose corticosteroids which could significantly influenced cellular immunity was a risk factor for CMV reactivation within 28 days from infusion. In addition, in a long follow-up, CD4+ T-cell count gradually recovered from infusion, and no one experienced a CD4+ T-cell count bellow 50/mL during observation. Only one patient developed CMV reactivation requiring preemptive therapy after day 28. She was treated with PSL for organizing pneumonia following severe acute respiratory syndrome coronavirus 2, and that decreased her CD4+ T-cell count from 92 to 61/µL. In contrast, B-cell aplasia continues for several years, and humoral immune deficiency is prolonged owing to the off-tumor effects of CAR T cells. Although some studies reported the importance of humoral immunity in CMV reactivation, the role of humoral immunity was less clear compared with cellular immunity [39, 40]. Even after allo-SCT, intravenous immunoglobulins cannot prevent CMV infection [41]. In this study, the IgG levels and frequency of intravenous immunoglobulin administration in the CMV reactivation group were equivalent to those in the non-CMV reactivation group. Furthermore, based on the long-term observation of 28 patients (28 days post-infusion), hypogammaglobulinemia persisted, and only one patient recovered from detectable B cell.

Currently, there is no established treatment for CMV reactivation in patients treated with CAR T-cell therapy.

Considering the rarity of CMV end-organ disease, routine preemptive therapy may be excessive. However, some reports have described severe CMV end-organ disease following CAR T-cell therapy [42–45]. These patients had some risk factors for CMV reactivation, such as primary refractory disease, extensive prior treatment, severe CRS/ICANS, and high-dose corticosteroid use. Because auto-SCT shows a similar frequency of CMV infection (30–50%) and CMV end-organ disease (< 1%) compared to CAR T-cell therapy, one report recommended aligning it with the institutional guidelines for auto-SCT [2]. When considering preemptive therapy, a pp65 antigen level ≥ 5 cells/slide is commonly used as the threshold for initiating preemptive treatment [15, 46].

In the present study, six patients were treated with ganciclovir or valganciclovir based on treating physician judgment, with more than number of positive cells of 6 cells/50,000 PBLs in pp65 antigenemia assays. In this study, threshold level of preemptive therapy almost was similar to that of auto-SCT, and none of the included patients experienced in CMV end-organ disease. However, whether this threshold is appropriate remains uncertain. Cytopenia are common adverse events associated with ganciclovir and valganciclovir [47], while CAR T-cell therapy also induces cytopenia [48]. Therefore, unnecessary preemptive therapy should be avoided. Further research is warranted to investigate the appropriate preemptive treatment of CMV reactivation based on monitoring with pp65 antigenemia assays.

This study had some limitations. First, this was a retrospective analysis from single institution, and the number of patients was relatively small. The majority of patients (91%) were treated with tisa-cel. Recently, CAR T-cell products with a CD28z co-stimulatory domain, such as axi-cel exhibit delayed CD4+ T-cell reconstitution than 4-1BBz co-stimulatory domain, such as tisa-cel and liso-cel [25]. Therefore, the incidence of CMV reactivation might increase over a longer follow-up period in patients treated with axi-cel. Second, no strict protocols existed for monitoring and treating CMV reactivation during the study period. Treatment decisions were made at the discretion of the attending physicians. Although physician judgment may have affected aspects such as the number of pp65 antigenemia assays performed or the treatment duration, we believe that this variability did not substantially affect the primary outcomes. Third, some patients lacked information regarding CMV seropositivity. In Japan, approximately 80–100% of adults are seropositive, which is consistent with our results (27 of 31 patients were seropositive, 87.1%) [6]. Fourth, a neutrophil count of 200/mL is usually required to perform the antigenemia assay [49]. In the present study, neutrophil counts fell below 200/mL in 65.2% patients. Although the duration of that was relatively short (median duration of 4.5 days), some cases of CMV reactivation may have been overlooked. Therefore, we do not recommend the pp65 assay over CMV-PCR, particularly for CMV-seropositive patients at high risk of CMV reactivation during the first 28 days post-infusion. In other clinical settings, the pp65 assay may remain a reasonable alternative if CMV-PCR is unavailable.

In conclusion, 19.6% of the patients monitored using pp65 antigenemia assay in this study developed CMV reactivation, none of whom developed CMV end-organ disease. Unless suppress the cellular immune system, CMV reactivation might be a rare event in patients more than 28 days post-infusion. In terms of sensitivity, the results should be carefully monitored, particularly during episodes of neutropenia. Our study showed usefulness of the monitoring of pp65 antigenemia assay in settings of CAR T-cell therapy. This study results would be helpful for patients in many countries which still use pp65 antigenemia assays for CMV reactivation monitoring. Determining the appropriate threshold for initiating preemptive therapy and whether the CAR-HEMATOTOX score could serve as a predictive marker for CMV reactivation will be important subjects for future research.

## Supplementary Information

Below is the link to the electronic supplementary material.

Supplementary file1 (DOCX 40 KB)

Supplementary file2 (JPG 1190 KB)

Supplementary file3 (JPG 2444 KB)

Supplementary file4 (JPG 2018 KB)

Supplementary file5 (JPG 976 KB)

Supplementary file6 (JPG 1005 KB)

Supplementary file7 (JPG 1030 KB)

## Data Availability

The datasets generated during and/or analyzed during the current study are available from Kenta Hayashino or Keisuke Seike on reasonable request.
