# Supplementary material for: Cytomegalovirus reactivation in patients with large B-cell lymphoma treated with chimeric antigen receptor T-cell therapy
Source: Int J Hematol. 2025 Jun 17;122(5):689–99. doi: 10.1007/s12185-025-04023-y (PMC12572091; doi:10.1007/s12185-025-04023-y)
Supplement: Supplementary file 1 — Supplementary file1 (DOCX 40 KB) [file 12185_2025_4023_MOESM1_ESM.docx]

**Supplemental Data**

**Title**

**Cytomegalovirus reactivation in patients with large B-cell lymphoma treated with chimeric antigen receptor T-cell therapy**

Kenta Hayashino^1,2^, Keisuke Seike^1^, Taro Masunari^3^, Risa Hashida^4^, Satoshi Oka^5^, Yuki Fujiwara^6^, Toshiki Terao^1,2^, Wataru Kitamura^1,2^, Hiroki Kobayashi^1,6^, Chihiro Kamoi^7^, Takumi Kondo^1^, Hideaki Fujiwara^1^, Noboru Asada^1^, Daisuke Ennishi^1,8^, Keiko Fujii^1,9^, Nobuharu Fujii^1,7^, Yoshinobu Maeda^1,2^

^1^ Department of Hematology and Oncology, Okayama University Hospital, 2-5-1 Shikata, Okayama-shi, Okayama, Japan

^2^ Department of Hematology, Oncology and Respiratory Medicine, Okayama University Graduate School of Medicine, Dentistry and Pharmaceutical Sciences, Okayama, Japan

^3^ Department of Hematology, Chugoku Central Hospital, 148-13, Oazakamiiwanari, Miyuki-cho, Fukuyama 720-0001, Japan

^4^ Division of Hematology, Ehime Prefectural Central Hospital, 83, Kasuga-machi, Matsuyama 790-0024, Japan

^5^ Department of Hematology and Blood Transfusion, Kochi Health Science Center, 2125-1, Ike, Kochi 781-8555, Japan

^6^ Department of Hematology and Oncology, Japanese Red Cross Society Himeji Hospital, 1-12-1, Shimoteno, Himeji 670-8540, Japan

^7^ Division of Transfusion and Cell Therapy, Okayama University Hospital, Okayama, 2-5-1 Shikata, Okayama-shi, Japan

^8^ Center for Comprehensive Genomic Medicine, Okayama University Hospital, 2-5-1 Shikata, Okayama-shi, Okayama, Japan

^9^ Division of Clinical Laboratory, Okayama University Hospital, Okayama, 2-5-1 Shikata, Okayama-shi, Japan

**Supplemental Table 1**

Univariate and multivariate analyses for overall survival

|  | Univariate | | | Multivariate | | |
| --- | --- | --- | --- | --- | --- | --- |
|  | HR | 95% CI | *P* value | HR | 95% CI | *P* value |
| CMV reactivation | 3.4 | 1.3-9.1 | 0.014 | 1.4 | 0.37-5.6 | 0.60 |
| Primary refractory | 2.5 | 1.0-6.3 | 0.043 | 1.7 | 0.51-5.9 | 0.39 |
| Number of prior regimens ≥ 4 | 4.3 | 1.3-14.9 | 0.020 | 3.5 | 0.97-12.9 | 0.056 |

CI, confidence interval; CMV, cytomegalovirus; HR, hazard ratio

**Supplemental Table 2**

Comparison of patient characteristics in CS-CMVi

|  | CS-CMVi  (n = 6) | Non- CS-CMVi  (n = 40) | *p* = |
| --- | --- | --- | --- |
| Median age, y (range) | 60.5 (53-69) | 58 (42-72) | 0.71 |
| Sex, n (%) |  |  | 1.0 |
| Male | 3 (50.0) | 18 (45.0) |  |
| Female | 3 (50.0) | 22 (55.0) |  |
| CMV-IgG, n (%) |  |  | 1.0 |
| Positive | 4 (66.7) | 24 (60.0) |  |
| Negative | 0 | 4 (10.0) |  |
| NA | 2 (33.3) | 12 (30.0) |  |
| Prior history of CMV infection, n (%) | 2 (33.3) | 4 (10.0) | 0.17 |
| Disease histology, n (%) |  |  | 0.58 |
| DLBCL-NOS | 5 (83.3) | 23 (57.5) |  |
| transformed | 1 (16.7) | 10 (25.0) |  |
| Other | 0 | 7 (17.5) |  |
| CAR-T products, n (%) |  |  | 1.0 |
| Tisa-cel | 6 (100) | 36 (90.0) |  |
| Liso-cel | 0 | 2 (5.0) |  |
| Axi-cel | 0 | 2 (5.0) |  |
| Prior history of auto-SCT, n (%) | 1 (16.7) | 18 (45.0) | 0.38 |
| Disease status, n (%) |  |  | 0.029 |
| CR | 0 | 9 (22.5) |  |
| primary refractory | 5 (83.3) | 10 (25.0) |  |
| relapse | 1 (16.7) | 21 (52.5) |  |
| Median number of prior regimens, n (range) | 4.5 (4-7) | 4.0 (2-6) | 0.025 |
| PS, n (%) |  |  | 0.079 |
| 0-1 | 3 (50.0) | 34 (85.0) |  |
| 2- | 3 (50.0) | 6 (15.0) |  |
| CAR-HEMATOTOX, n (%) |  |  | 0.035 |
| High | 6 (100) | 21 (52.5) |  |
| Low | 0 | 19 (47.5) |  |
| Median LDH (U/L) (range) | 345.0 (217-1344) | 227 (136-1250) | 0.0090 |
| Median Ly (/μL) (range) | 345 (150-700) | 525 (100-2150) | 0.10 |
| Median B-cell (/μL) (range) | 0 (0-0) | 0 (0-0) | NA |
| Median CD4+T-cell (/μL) (range) | 147 (45-482) | 102 (102-102) | 0.66 |
| Median CD8+T-cell (/μL) (range) | 563 (74-759) | 163 (163-163) | 0.66 |
| Lymphodepleting chemotherapy, n (%) |  |  | 0.20 |
| FLU/CY | 5 (83.3) | 36 (90.0) |  |
| Other | 1 (16.7) | 4 (10.0) |  |
| CRS, n (%) |  |  | 0.015 |
| Grade 0-1 | 1 (16.7) | 29 (72.5) |  |
| Grade 2-4 | 5 (83.3) | 11 (27.5) |  |
| ICANS, n (%) |  |  | 1.0 |
| Grade 0-1 | 6 (100) | 36 (90.0) |  |
| Grade 2-4 | 0 | 4 (10.0) |  |
| Corticosteroid use, n (%) | 4 (66.7) | 26 (65.0) | 1.0 |
| Cumulative dose of PSL > 1000mg, n (%) | 3 (50.0) | 3 (7.5) | 0.079 |
| Tocilizumab use, n (%) | 6 (100) | 33 (82.5) | 0.57 |
| Cumulative dose of tocilizumab, median (range) | 1.5 (1-4) | 2 (0-4) | 0.58 |

Axi-cel, axicabtagene ciloleucel; auto-SCT, autologous stem cell transplantation, CAR-T, chimeric antigen receptor-T; CMV, cytomegalovirus; CR, complete response; CRS, cytokine release syndrome; CS-CMVi, clinically significant cytomegalovirus infection; CY, cyclophosphamide; DLBCL-NOS, diffuse large B-cell lymphoma-not otherwise specified; FLU, fludarabine; ICANS, immune effector cell-associated neurotoxicity syndrome; LDH, lactate dehydrogenase; Liso-cel, lisocabtagene maraleucel; Ly, lymphocyte; NA, not available; PS, performance status; PSL, prednisolone; Tisa-cel, tisagenlecleucel

**Supplemental Table 3**

Univariate analysis of risk factor for CS-CMVi.

|  | HR | 95% CI | P value |
| --- | --- | --- | --- |
| Age > 60 | 1.5 | 0.32-7.2 | 0.60 |
| Prior history of CMV reactivation | 5.5 | 0.98-30.3 | 0.051 |
| Prior history of auto-SCT | 0.21 | 0.023-1.9 | 0.16 |
| Primary refractory | 14.1 | 1.7-115.4 | 0.014 |
| Number of prior regimens > 4 | 3.3 | 0.73-15.4 | 0.12 |
| LDH > ULN | 3.3 | 0.41-26.5 | 0.26 |
| Lymphocyte counts < 500/µL | 2.2 | 0.44-11.3 | 0.33 |
| CRS Grade 2-4 | 9.5 | 1.1-82.6 | 0.042 |
| Use of PSL > 1000mg | 10.8 | 1.9-60.1 | 0.0068 |
| IgG day0-28 < 500mg/dL | 4.3 | 0.84-21.9 | 0.081 |

auto-SCT, autologous stem cell transplantation; CI, confidence intervals; CMV, cytomegalovirus; CRS, cytokine release syndrome; CS-CMVi, clinically significant cytomegalovirus infection; HR, hazard ratio; IgG, immunoglobulin G; LDH, lactate dehydrogenase; PSL, prednisolone; sIL2R, soluble interleukin-2 receptor; ULN, upper limit of normal

**Supplemental Table 4**

Characteristics of patients followed for more than 28 days

|  | Long follow cohort (n = 28) |
| --- | --- |
| Median age, y (range) | 58 (45-72) |
| Sex, n (%) |  |
| Male | 16 (57.1) |
| Female | 12 (42.9) |
| CMV-IgG, n (%) |  |
| Positive | 18 (64.3) |
| Negative | 3 (10.7) |
| NA | 7 (25.0) |
| Prior history of CMV infection, n (%) | 3 (10.7) |
| Disease histology, n (%) |  |
| DLBCL-NOS | 16 (57.1) |
| transformed | 8 (28.6) |
| other | 4 (14.3) |
| CAR-T products, n (%) |  |
| Tisa-cel | 27 (96.4) |
| Liso-cel | 1 (3.6) |
| Axi-cel | 0 |
| Prior history of auto-SCT, n (%) | 11 (39.3) |
| Disease status, n (%) |  |
| CR | 6 (21.4) |
| primary refractory | 8 (28.6) |
| relapse | 14 (50.0) |
| Median No. of prior regimens, n (range) | 4 (2-6) |
| PS, n (%) |  |
| 0-1 | 24 (85.7) |
| 2- | 4 (14.3) |
| CAR-HEMATOTOX, n (%) |  |
| High | 16 (57.1) |
| Low | 12 (42.9) |
| Median LDH (range) (U/L) | 227 (136-634) |
| Median Ly (range) (/μL) | 515 (250-2150) |
| Median B-cell (range) (/μL) | 0 (0-0) |
| Median CD4+T-cell (range) (/μL) | 205 (45-482) |
| Median CD8+T-cell (range) (/μL) | 203 (74-632) |
| Lymphodepleting chemotherapy, n (%) |  |
| FLU/CY | 27 (96.4) |
| Other | 1 (3.6) |
| CRS, n (%) |  |
| Grade 0-1 | 17 (60.7) |
| Grade 2-4 | 11 (39.3) |
| ICANS, n (%) |  |
| Grade 0-1 | 28 (100) |
| Grade 2-4 | 0 |
| Corticosteroid use, n (%) | 19 (67.9) |
| Cumulative dose of PSL > 1000mg, n (%) | 5 (17.9) |
| Tocilizumab use, n (%) | 23 (82.1) |
| Cumulative dose of tocilizumab, median (range) | 2 (0-4) |
| Number of pp65 antigenemia assays, median (range) | 7 (2-42) |

Axi-cel, axicabtagene ciloleucel; auto-SCT, autologous stem cell transplantation, CAR-T, chimeric antigen receptor-T; CMV, cytomegalovirus; CR, complete response; CRS, cytokine release syndrome; CY, cyclophosphamide; DLBCL-NOS, diffuse large B-cell lymphoma-not otherwise specified; FLU, fludarabine; ICANS, immune effector cell-associated neurotoxicity syndrome; LDH, lactate dehydrogenase; Liso-cel, lisocabtagene maraleucel; Ly, lymphocyte; NA, not available; PS, performance status; PSL, prednisolone; Tisa-cel, tisagenlecleucel

**Supplemental Figure Legends**

**Supplemental Fig. 1**

The kinetics of neutrophil (median ± IQR) in before and after CAR T-cell infusion.

CAR T, chimeric antigen receptor T; IQR, interquartile range

**Supplemental Fig. 2**

Comparison of survival outcomes between CMV reactivation and non-CMV reactivation group. Patients in CMV reactivation group had worse PFS (a) and OS (b)

CAR T, chimeric antigen receptor T; CMV, cytomegalovirus; OS, overall survival; PFS, progression free survival

**Supplemental Fig. 3**

The cumulative incidence of CS-CMVi

CAR T, chimeric antigen receptor T; CS-CMVi, clinically significant cytomegalovirus infection

**Supplemental Fig. 4**

The kinetics of IgG (a), CD4T-cell (b), CD8T-cell (c) (median ± IQR) levels in long-term follow-up cohort.

CAR T, chimeric antigen receptor T; IgG, immunoglobulin G; IQR, interquartile range
