# Supplementary figures and images for: Cytomegalovirus reactivation in patients with large B-cell lymphoma treated with chimeric antigen receptor T-cell therapy
Source: Int J Hematol. 2025 Jun 17;122(5):689–99. doi: 10.1007/s12185-025-04023-y (PMC12572091; doi:10.1007/s12185-025-04023-y)

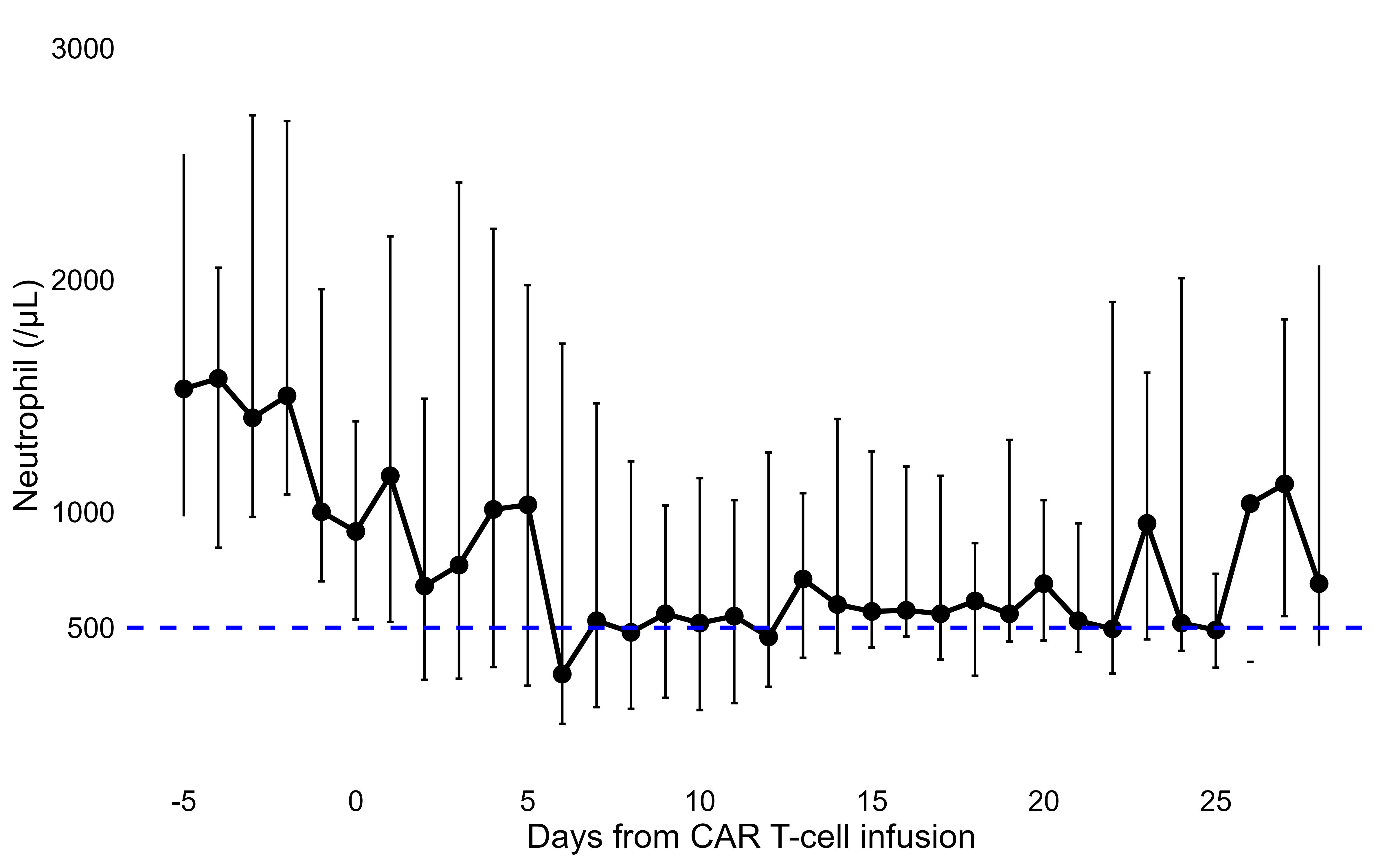

Supplement: Supplementary file 2 — Supplementary file2 (JPG 1190 KB) [file 12185_2025_4023_MOESM2_ESM.jpg]

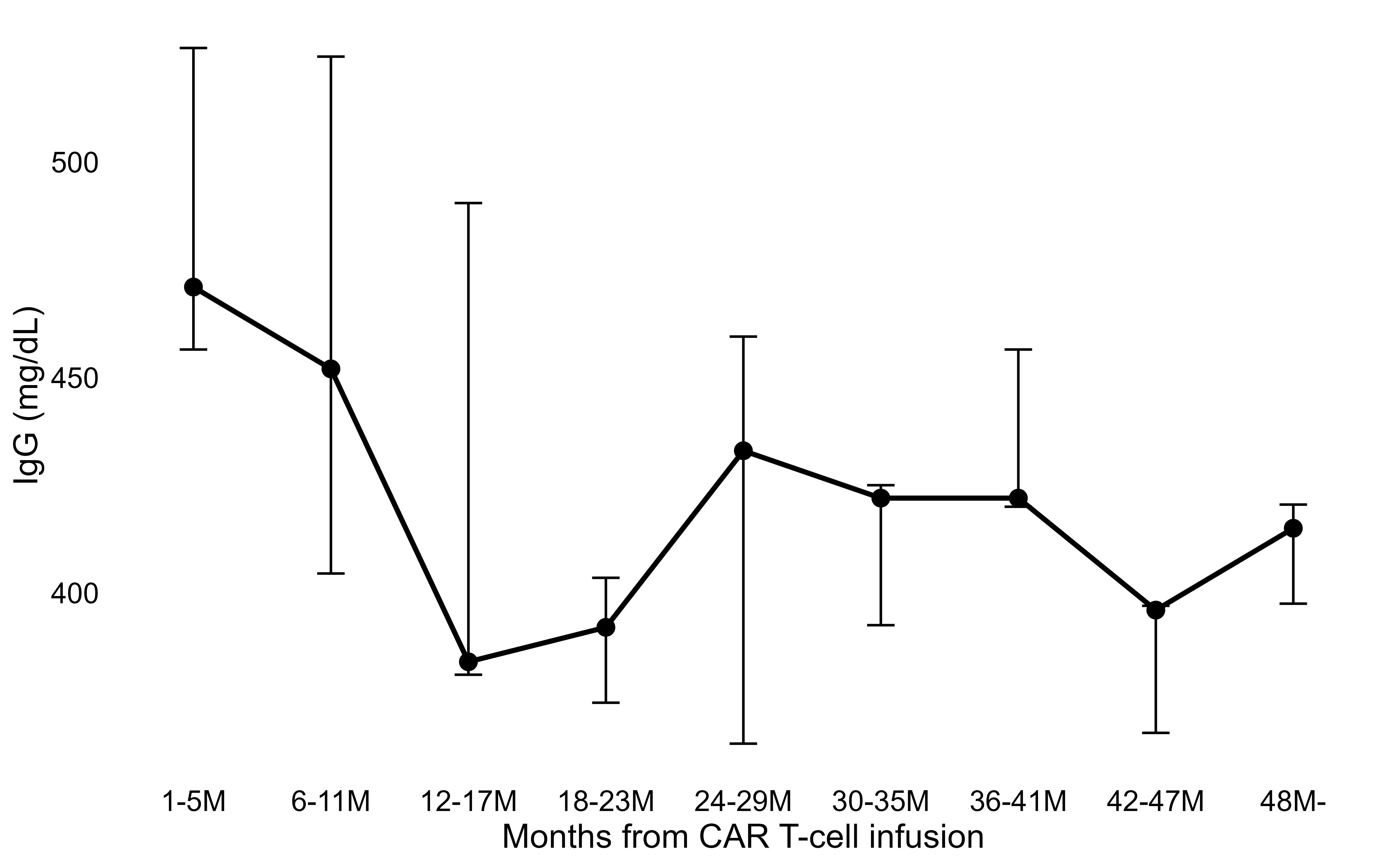

Supplement: Supplementary file 5 — Supplementary file5 (JPG 976 KB) [file 12185_2025_4023_MOESM5_ESM.jpg]

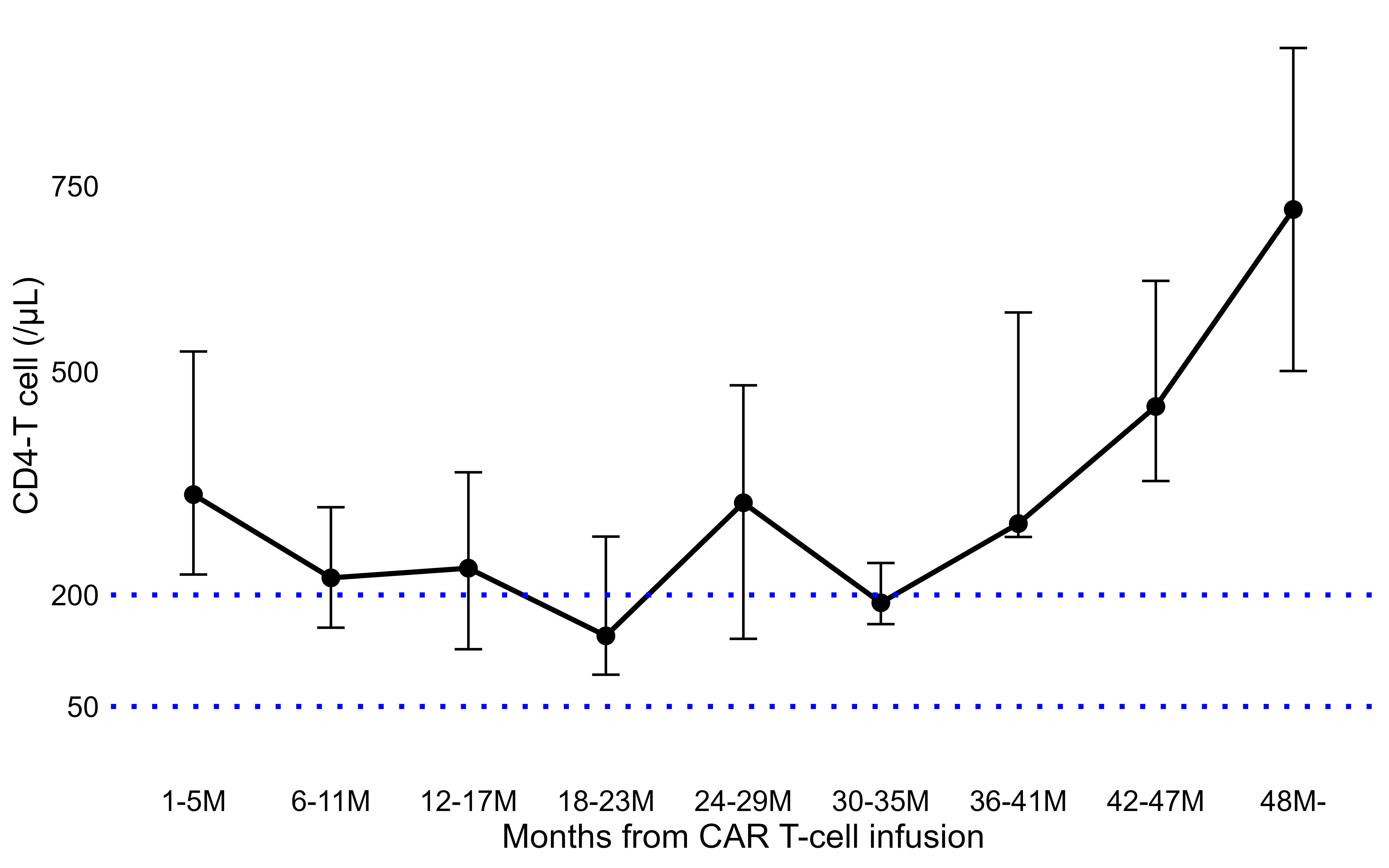

Supplement: Supplementary file 6 — Supplementary file6 (JPG 1005 KB) [file 12185_2025_4023_MOESM6_ESM.jpg]

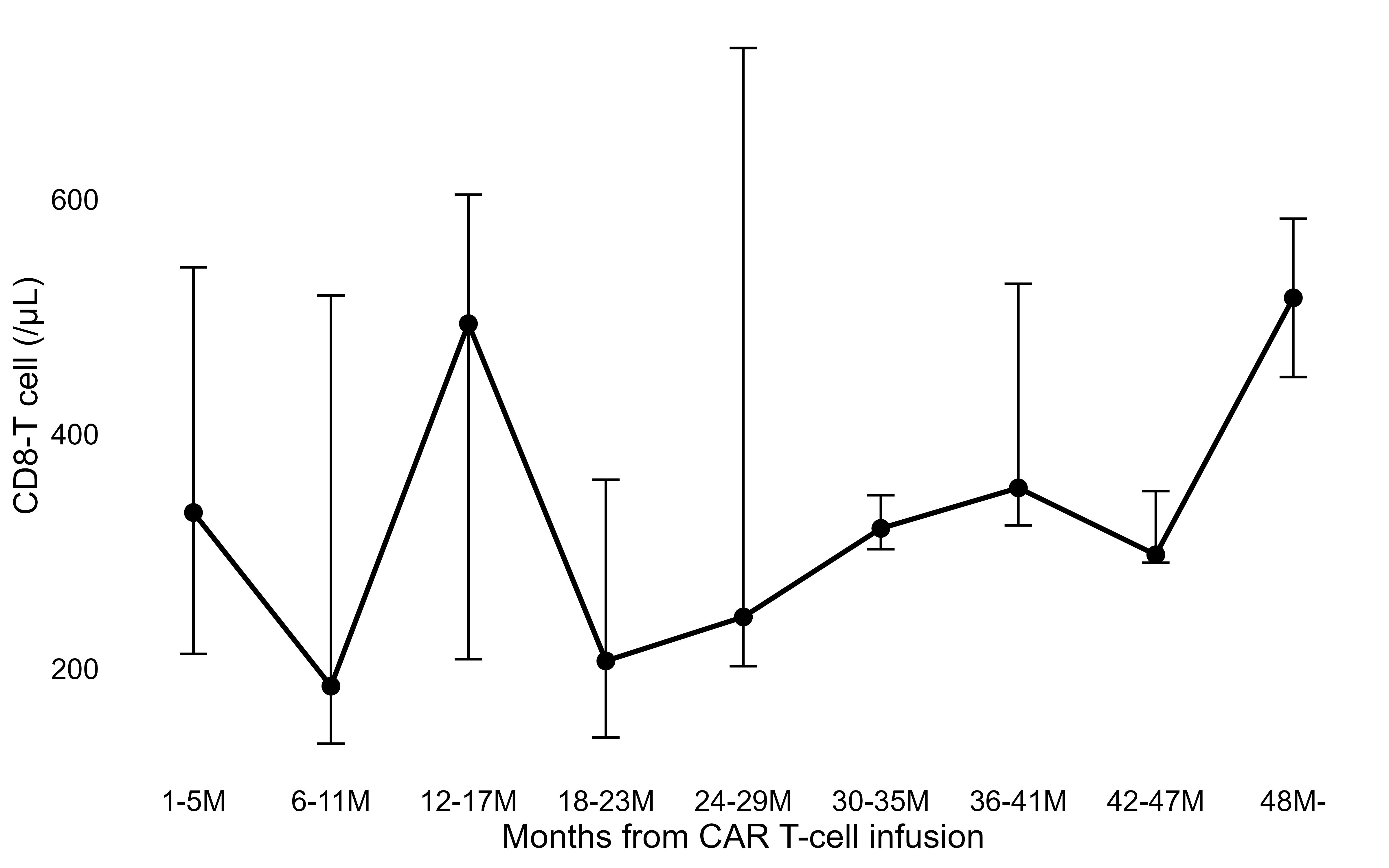

Supplement: Supplementary file 7 — Supplementary file7 (JPG 1030 KB) [file 12185_2025_4023_MOESM7_ESM.jpg]
